# Portable Optical Fiber Probe-Based Spectroscopic Scanner for Rapid Cancer Diagnosis: A New Tool for Intraoperative Margin Assessment

**DOI:** 10.1371/journal.pone.0030887

**Published:** 2012-01-27

**Authors:** Niyom Lue, Jeon Woong Kang, Chung-Chieh Yu, Ishan Barman, Narahara Chari Dingari, Michael S. Feld, Ramachandra R. Dasari, Maryann Fitzmaurice

**Affiliations:** 1 George R. Harrison Spectroscopy Laboratory, Massachusetts Institute of Technology, Cambridge, Massachusetts, United States of America; 2 Optics Research Laboratory, Research & Development Division, Canon U. S. A., Inc., Tucson, Arizona, United States of America; 3 Department of Pathology, Case Western Reserve University, Cleveland, Ohio, United States of America; University of Queensland, Australia

## Abstract

There continues to be a significant clinical need for rapid and reliable intraoperative margin assessment during cancer surgery. Here we describe a portable, quantitative, optical fiber probe-based, spectroscopic tissue scanner designed for intraoperative diagnostic imaging of surgical margins, which we tested in a proof of concept study in human tissue for breast cancer diagnosis. The tissue scanner combines both diffuse reflectance spectroscopy (DRS) and intrinsic fluorescence spectroscopy (IFS), and has hyperspectral imaging capability, acquiring full DRS and IFS spectra for each scanned image pixel. Modeling of the DRS and IFS spectra yields quantitative parameters that reflect the metabolic, biochemical and morphological state of tissue, which are translated into disease diagnosis. The tissue scanner has high spatial resolution (0.25 mm) over a wide field of view (10 cm×10 cm), and both high spectral resolution (2 nm) and high spectral contrast, readily distinguishing tissues with widely varying optical properties (bone, skeletal muscle, fat and connective tissue). Tissue-simulating phantom experiments confirm that the tissue scanner can quantitatively measure spectral parameters, such as hemoglobin concentration, in a physiologically relevant range with a high degree of accuracy (<5% error). Finally, studies using human breast tissues showed that the tissue scanner can detect small foci of breast cancer in a background of normal breast tissue. This tissue scanner is simpler in design, images a larger field of view at higher resolution and provides a more physically meaningful tissue diagnosis than other spectroscopic imaging systems currently reported in literatures. We believe this spectroscopic tissue scanner can provide real-time, comprehensive diagnostic imaging of surgical margins in excised tissues, overcoming the sampling limitation in current histopathology margin assessment. As such it is a significant step in the development of a platform technology for intraoperative management of cancer, a clinical problem that has been inadequately addressed to date.

## Introduction

Fast and reliable intraoperative tissue diagnosis is a critical component of successful cancer surgery in a variety of organ systems. Yet there continues to exist a significant clinical need for rapid and reliable intraoperative margin assessment of excised surgical specimens. Currently, intraoperative margin assessment is done by visual inspection and palpation, followed by selective assessment of any suspicious areas by rapid histology or cytology evaluation, which can be time consuming and inaccurate due to limited sampling. It is not unusual for the result of this pathologic margin assessment to come after the surgical wound is closed and the patient moved to the recovery room. Further, there are frequent discrepancies between the selective intraoperative and more comprehensive postoperative pathology margin assessment, necessitating reoperation to achieve negative margins. In breast-conserving surgery, for example, re-operation for positive margins discovered after surgery is required in up to 50% of cases [1]. Furthermore, breast cancer recurs locally in the surgical bed in ∼10% of patients with negative margins on postoperative pathology margin assessment [2] which, while more comprehensive than intraoperative pathology assessment, is still subject to sampling limitations. Intraoperative assessment of surgical margins is, thus, an important step in surgical management of cancer that has been inadequately addressed to date. The challenge in addressing this need is to develop an imaging system with a wide enough field of view to image large surgical specimens with high enough resolution to detect small foci of cancer at the surgical margin in a clinically useful time frame.

Optical fiber probe-based diffuse reflectance spectroscopy (DRS) and intrinsic fluorescence spectroscopy (IFS) are being actively pursued as tools for the real time diagnosis of cancer [3], and have advantages over other approaches to intraoperative and comprehensive assessment of surgical margins. DRS and IFS depend on the inherent optical properties of tissue and, as such, do not require exogenous imaging probes or contrast agents. The combination of DRS and IFS provides information on the metabolic, biochemical and morphological state of tissue, which can be translated into disease diagnosis. DRS and IFS have relatively shallow (≤1 mm) tissue penetration, and thus interrogate only the margin of the excised tissue specimen. Unlike traditional pathology diagnosis, spectroscopic diagnosis can be performed in real time. Spectroscopic techniques are also quantitative and therefore more objective than the traditional approach, which is subject to pathologist interpretation. However, conventional optical fiber probe-based spectroscopy techniques only examine a small area of tissue (∼1 mm) at a time, and so can suffer from under sampling and easily miss the lesion of interest. Spectroscopic imaging techniques [4]–[9] can examine the entire margin of the excised tissue specimen, and so are not prone to the sampling limitations inherent in traditional pathology examination.

Here we present a portable, quantitative, optical fiber probe-based, spectroscopic tissue scanner that can provide real-time comprehensive assessment of surgical margins in excised tissue specimens. The scanner significantly advances our optical fiber probe-based spectroscopy instruments [10], [11], which have been successfully employed in clinical studies for the diagnosis of oral, esophageal, cervical and breast cancer [12]–[15], to a wide field, high resolution imaging regime as required to be an effective clinical tool for intraoperative margin assessment at cancer surgery. This tissue scanner is simpler in design, images a larger field of view at higher resolution and provides a more physically meaningful tissue diagnosis than other spectroscopic margin imaging systems currently in development. Overall, the tissue scanner can provide fast, accurate, diagnostic images of the entire margin of excised surgical specimens, overcoming the sampling limitation in current pathology margin assessment. We believe the tissue scanner is a platform technology that has the potential to provide real-time, comprehensive, intraoperative assessment of surgical margins that will allow more complete resection of diseased tissue and better conservation of normal tissue at surgery for breast cancer and other solid tumors.

## Materials and Methods

### Instrumentation

**Figure 1:**
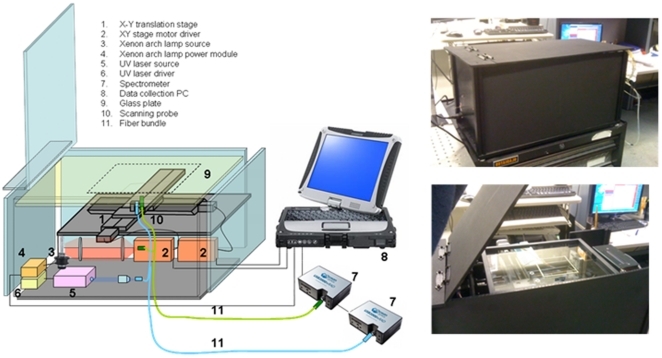
Tissue Scanner. Schematic diagram of the tissue scanner and photographs of the unit from different views.

A portable tissue scanner was constructed that can scan large tissue specimens (up to 20 cm×20 cm) at high resolution (0.25 mm) in a clinically acceptable time frame (less than 20 minutes for 8 cm×8 cm area and 0.25 mm resolution). Figure 1 shows a schematic diagram and photographs of the tissue scanner unit. The scanner employs unitary multimodal optical fiber probes that we have successfully employed in a multimodal clinical spectroscopy system [10] for point spectroscopy measurements. Two optical fiber probes are used, one for DRS and another for IFS, at a fixed separation of 0.75 cm to minimize cross talk between two probes. Each probe consists of a fiber bundle with a single central fiber that delivers excitation light to the tissue, surrounded by a ring of multiple fibers that collect reflected and fluorescent light returning from the sample and transmit it to the spectrograph (all fibers have 200 µm core and NA = 0.22), terminated with a transparent, protective optical shield. In this study, only one of the collection fibers was used in each probe. A 75 W Xenon arc lamp (Oriel Instrument, USA) is used to generate excitation light for DRS and a 7 mW Q-switched solid state laser at 355 nm (SNV-40F-000, Teem Photonics) to generate excitation light for IFS. This wavelength was selected based on the previous IFS studies of breast cancer [15]. However, this is a platform technology that can easily be used with other excitation wavelengths for other diagnostic applications. Signals are collected with miniature spectrometers (USB2000+, Ocean Optics). The spectrometers have spectral resolution of 2 nm at full width half maximum (FWHM). The wide area imaging capability is achieved by mechanically scanning optical probes with a long traveling range, XY translation stage and step motors (Applied Motion Products, micro stepper motor: 17-075 and driver: 3540i) in an inverted geometry through a standard glass plate (20 cm×30 cm×0.16 cm) on which the specimen rests. There is no interference from glass fluorescence with the biomolecular fluorophores of interest: collagen and NADH. The glass plate flattens the tissue surface and provides a reasonably uniform probe-tissue imaging distance. This allows us to make quantitative measurements, by preserving the key optical characteristics of the probe (spot size and NA), and take full advantage of our clinically proven, probe-based spectroscopic models [12]–[15], which would not be applicable to data acquired with a free space imaging system. Excitation beam spot size at the surface of a tissue sample sitting on the glass plate is estimated to be <1 mm. Labview 8.6 (National Instrument, TX) manages the raster scan by commanding the XY stage through PC serial ports and the spectral data acquisitions. Total scanning time for the tissue sample depends on the choice of parameters such as excitation power, integrating time, spatial resolution, field of view, etc., which can be adjusted according to tissue type and clinical need. Note that total scanning time includes timing response to start/stop and reverse the stepper motors. The portable device measures 60 cm×30 cm×30 cm, weighs 13.6 kg and can easily fit in most clinical spaces including patient examination rooms, procedure rooms and operating rooms.

### Data processing

DRS and fluorescence spectra (350–700 nm) are obtained for each spot scanned. After background subtraction and normalization with 20% Spectralon white reflectance standards (Labsphere, NH), DRS spectra are analyzed using a mathematical model based on the diffusion approximation of light propagation in tissue [16]. IFS spectra are then obtained, by correcting the raw fluorescence spectra for the effects of tissue absorption and scattering using the corresponding DRS spectra [17]–[18], and analyzed using a linear combination model based on multivariate curve resolution (MCR), a standard chemometric method [15]. Spectral modeling provides physically meaningful fitting parameters that are quantitative measures the contributions of specific tissue components. These spectral parameters are the basis of decision algorithms used in the diagnosis of breast [15] and other cancers [12]–[14]. DRS modeling yields 3 scattering parameters: A, which is related to the amount of Mie scatterers; B, which is related to the size of the scatterers; and C, which is related to the amount of Rayleigh scatterers; and absorption fitting parameters for hemoglobin (Hb) and β-carotene, two well-characterized absorbers in breast tissue. IFS modeling yields fluorescence fitting parameters related to NADH, a cellular metabolite, and collagen, a fluorophore that is more abundant in the fibrous stroma of breast cancer than in normal breast tissue.

DRS and IFS data cubes, i.e. three dimensional arrays of image XY coordinates and wavelength, are obtained during each scan. The overlap regions are then co-registered, without the need for complex mathematical transformation except simple shifting of the XY register in acquired pixels, which was previously obtained from the relative position of the probe during calibration. By registration of DRS and IFS probe positions, we can easily reassemble 2D quantitative hyperspectral DRS and IFS intensity maps of the scanned tissue surface. The spectra are then modeled and spectral fitting parameters extracted on a pixel-by-pixel basis to create quantitative parameter maps. A tissue diagnosis can also be rendered using a parameter-based decision algorithm to create diagnostic maps. Specifically, for the breast tissue studies, a previously developed DRS-IFS diagnostic algorithm [15] was applied to the fitting parameters on a pixel-by-pixel, and each pixel assigned a diagnosis of normal breast or breast cancer, to build a false color diagnostic map of the scanned tissue surface. Currently data is processed off line, and can take up to a second per data point for model fitting. We envision that real-time (on-the-fly) data processing can be performed by incorporating cutting edge machine learning algorithms that have been recently investigated for spectroscopic modalities by several laboratories including our own [19]–[22].

### Tissue simulating phantoms

Tissue-simulating liquid phantoms were prepared from various mixtures of intralipid (Invitrogen), hemoglobin (Hb) (Sigma Aldrich) or blood, and furan (Sigma Aldrich) to validate quantitative extraction of tissue absorption and florescence properties from the spectral data obtained with the scanner. Self-adhesive O-rings with an internal diameter of 1 cm were arranged on the glass plate to hold droplets of the liquid phantoms in place. Using a microliter pipette, 200 µL of each liquid phantom was carefully placed into the O-rings, forming droplets ∼2.5 mm in depth. Spectralon standards (10% and 20%) were also placed in the field of view, and were used to normalize the spectral data. Spectra obtained from each spot within each phantom were averaged.

### Animal tissue

An animal tissue study was performed to demonstrate high resolution wide-field hyperspectral imaging capability and spectral contrast to distinguish tissue structures with varying optical properties. As the animal tissue used was obtained from a commercial source, a grocery store (Shaw's Supermarket, Lynn MA), the investigator had no direct or indirect control over pre-mortem procedures or euthanasia, and the occupational health risks are nil, the Institutional Animal Care and Use Committee (IACUC) at Massachusetts Institute of Technology and at Case Western Reserve University did not require a protocol. A clean-cut cross section of an unfixed, frozen-thawed porcine lower leg tissue specimen (∼10 cm in diameter) was used. Before placing the tissue section on the glass plate for scanning, it was moistened with normal saline. Porcine lower leg tissue was used for this study as it has more anatomic detail requiring high resolution imaging and a wider range of tissue types with more varied optical properties than breast tissue.

### Human breast tissues

A human tissue study was performed to demonstrate the capability of the tissue scanner for breast cancer diagnosis. The study was conducted under a discarded tissue protocol with a waiver of informed patient consent approved by the University Hospitals Case Medical Center Institutional Review Board and MIT Committee on the Use of Humans as Experimental Subjects. A paired set of unfixed, frozen breast tissues, one grossly cancerous and another matched grossly normal breast tissue from the same patient, were obtained from the Case Comprehensive Cancer Center Human Tissue Procurement Facility for scanning on the tissue scanner. The tissues were shipped frozen on dry ice and thawed at room temperature before scanning. To prevent dehydration, the tissues were moistened with a small amount of normal saline. The tissues were placed side-by-side on the scanner glass plate. After scanning, the tissue surfaces scanned were marked with colored colloidal inks to preserve orientation, fixed in 10% neutral buffered formalin, processed and embedded in paraffin, and hematoxylin and eosin stained tissue sections prepared for microscopic examination by an experienced breast pathologist at the University Hospitals Case Medical Center, for comparison with the spectroscopic imaging results.

## Results

A number of studies were performed to test system performance. A resolution target was used to demonstrate the imaging capability and to test spatial resolution of the system. A series of tissue-simulating liquid phantoms was used to validate quantitative extraction of tissue properties from the reflectance and fluorescence spectral data. Moreover, animal tissue was used to illustrate the hyperspectral imaging capability of the system and demonstrate spectral contrast to distinguish tissues with different scattering and absorption properties. Finally, normal and cancer tissues from a breast cancer patient were used to demonstrate the usefulness of the tissue scanner as a cancer margin assessment device.

### Spatial resolution

**Figure 2:**
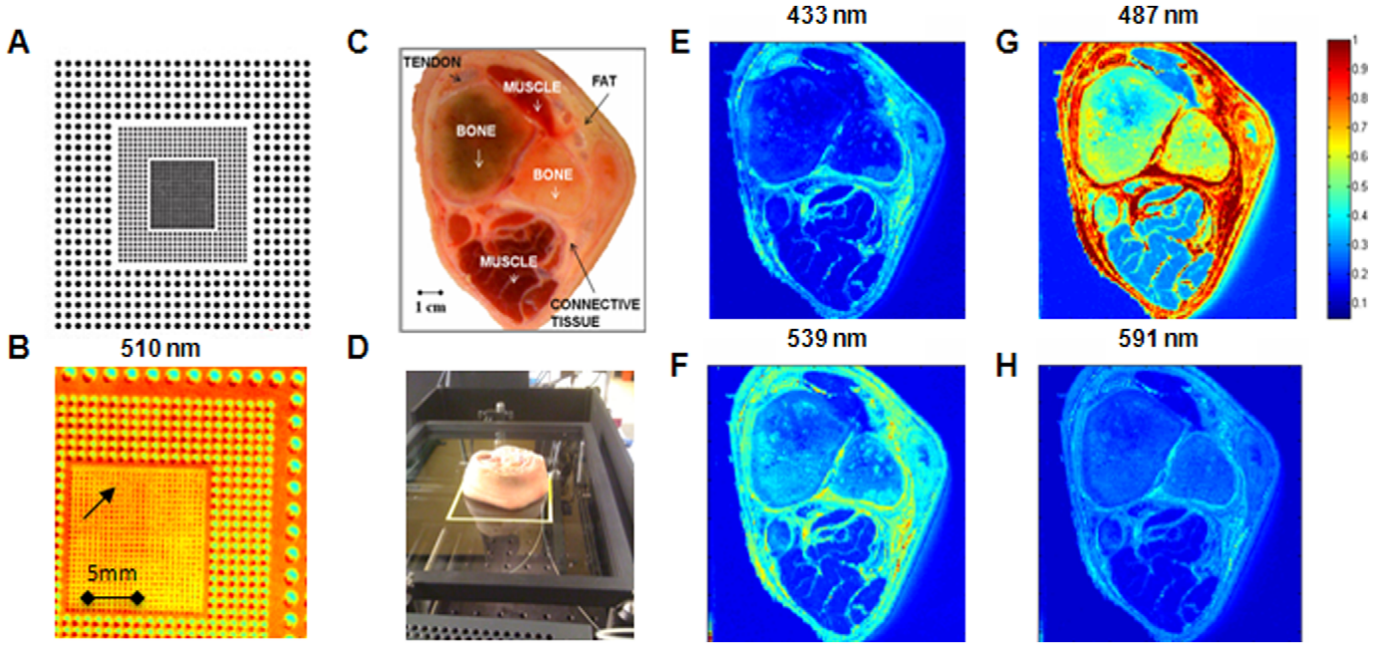
Spatial Resolution and Spectral Contrast. A photo of the resolution target (A) and a randomly selected 2D 2.5 cm×2.5 cm DRS spectral intensity map of zoomed target at 510 nm (B); a gross photograph of the porcine lower leg tissue cross section that was scanned (C); a photograph of the tissue cross section on the glass plate during scanning (D); and 9 cm×9 cm DRS images of the tissue cross section at different wavelengths (E–H). Note that the color bar is for all DRS images and is in arbitrary units.

In the DRS resolution target experiment, a positive multi-frequency grid distortion target (NT46-250, Edmond Optics, Figure 2A) was placed face-down on the glass plate and a DRS scan obtained from a 2.5 cm×2.5 cm area with excitation power 200 mW, integrating time 10 milliseconds, spatial or image pixel resolution 0.25 mm. Total acquisition time for the DRS image was less than 10 minutes. At 500 nm wavelength, the smallest dot features printed in the target, 250 microns in diameter, can be resolved as shown in the randomly selected DRS image at 510 nm in Figure 2B. Similar resolution was also found for IFS scanning. In this experiment, a droplet of furan (0.8 µg/mL) and intralipid (1%) was placed inside an O-ring on the glass plate, a fluorescence line scan acquired across the droplet, and the edge response [23] measured at 425 nm, yielding an IFS resolution of 250 microns (10% to 90% transition). These studies show that the tissue scanner can image a large field of view with sub-millimeter resolution.

### Field of view and spectral contrast

Next, a clean-cut cross section of a frozen-thawed porcine lower leg tissue specimen (∼10 cm in diameter) was used to demonstrate scanning of large fields of view and spectral contrast to distinguish tissue structures with different optical properties (Figure 2C). After placement on the glass plate (Figure 2D), DRS scans were performed with excitation power 250 mW, integrating time 50 milliseconds and spatial resolution 0.5 mm. Total acquisition time for the DRS image was 30 minutes. Figure 2E shows DRS spectral intensity maps acquired with the scanner at selected wavelengths. The DRS spectral intensity map at 487 nm was optimal for distinguishing tissue types with widely differing optical properties in this tissue, including bone, skeletal muscle, fat and connective tissues. This biological tissue study shows that the tissue scanner can image a large field of view with both high spatial resolution and spectral contrast to distinguish tissues with differing optical properties.

### Calibration and validation of quantitative measurements

**Figure 3:**
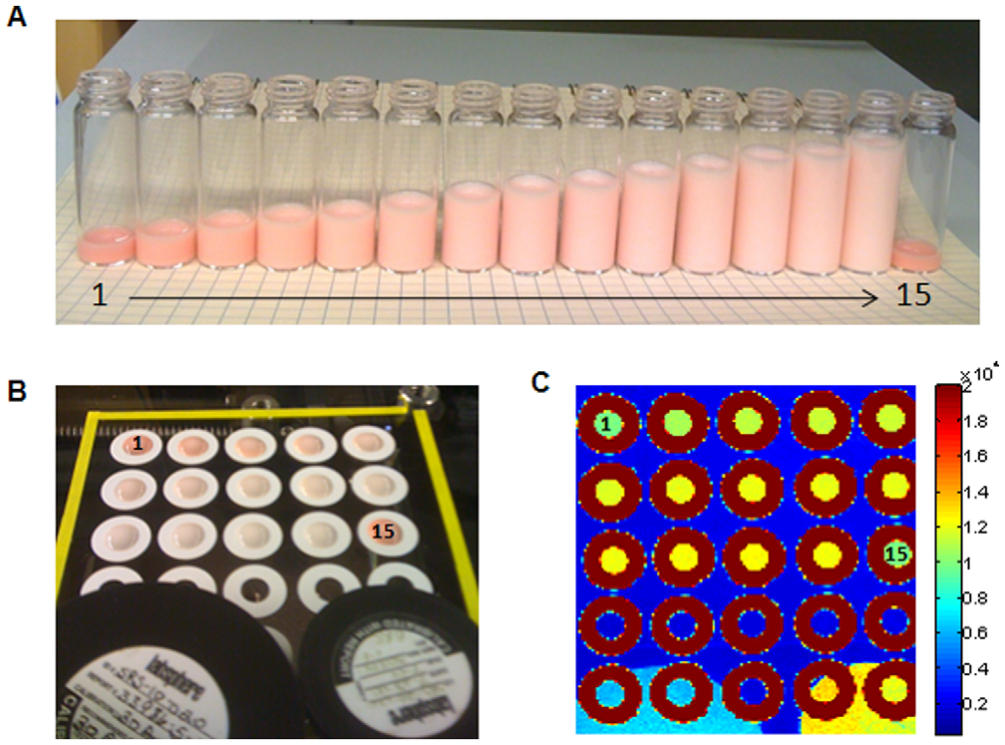
DRS Tissue Simulating Phantoms. Fifteen liquid phantoms composed of 2% intralipid and varying Hb concentrations in glass vials (A); O-rings filled with liquid phantoms and Spectralon standards (10% and 20%) on the glass plate, with the scanning field of view marked with yellow tape (B); 2D DRS scan of the phantoms and Spectralon standards at 490 nm (field of view = 10 cm×10 cm) (C).

**Figure 4:**
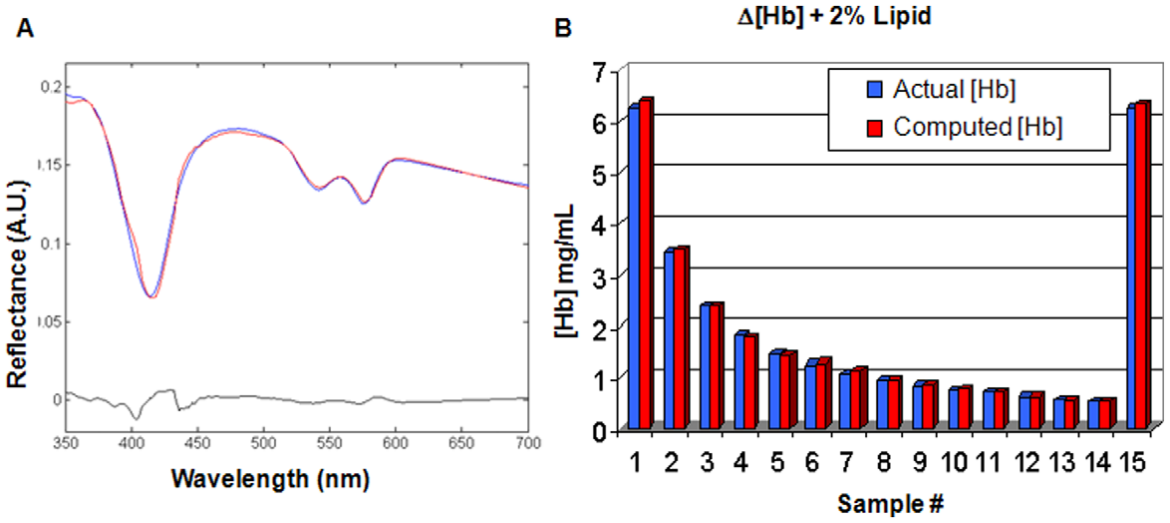
Validation of Hb Quantitation in DRS Tissue Simulating Phantoms. DRS spectra from a liquid phantom with 1.8 mg/ml Hb (blue), corresponding model fit (red) and residual (black) (A); Bar chart showing the Hb concentration curve results (B).

Fifteen liquid tissue simulating phantoms, composed of a 2% intralipid solution and various amounts of Hb powder to simulate breast tissue scattering and absorption, were prepared and used for DRS calibration. DRS scans were performed with excitation power 150 mW, integrating time 100 milliseconds and spatial resolution 0.5 mm. Hb concentration is highest in the first phantom (#1) and last phantom (#15), and progressively decreases from phantom 1 to phantom 14. A 2D DRS scanned image of the phantoms and Spectralon standards at a randomly selected wavelength (490 nm) is shown in Figure 3C. As expected, the reflectance signal intensity was inversely proportional to Hb concentration (due to Hb absorption). That is, the reflectance signal is the lowest in phantoms #1 and #15, and increases with decreasing Hb concentration from phantom #1 to phantom #14. Figure 4A shows a DRS spectrum of a phantom with 1.8 mg/ml Hb along with its corresponding model fit and residual. Figure 4B shows Hb concentration computed from the average phantom DRS spectra, which show excellent agreement with the actual phantom Hb concentrations (error≤5%). All calculated scattering-related parameters were constant in all samples. Additional experiments with constant Hb concentration and varying intralipid scatterer concentration showed the computed scattering parameters were proportional to intralipid concentration (data not shown). The results of these liquid phantom experiments confirm that the tissue scanner can accurately measure a physiologically relevant range of DRS absorption and scattering parameters across a large scanning field of view.

**Figure 5:**
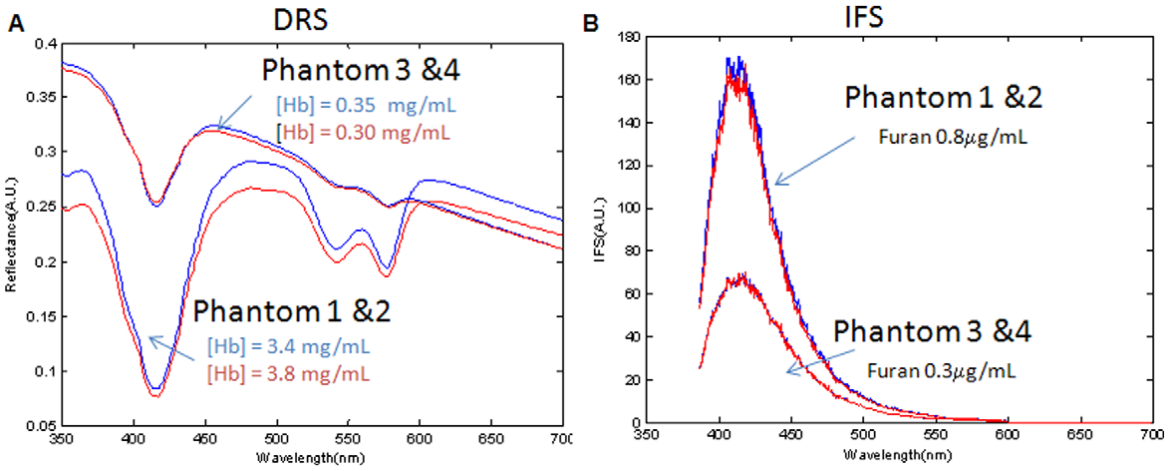
Validation of Fluorescence Measurements in IFS Tissue Simulating Phantoms. DRS (a) and IFS spectra(b) of phantoms with varying furan concentration (phantom 1 and 2 furan = 0.8 µg/mL); phantom 3 and 4 furan = 0.3 µg/mL).

For IFS validation, 4 liquid tissue simulating phantoms were used, consisting of two replicates each of a 1% intralipid solution with two different concentrations of human blood and furan (0.3 and 0.8 µg/mL), which has strong fluorescence in the 400–450 nm region (Table 1). Note that a pin-prick blood sample was used instead of Hb powder to provide additional biochemical fluorophores and scatterers with which to test DRS correction of the fluorescence spectra for the effects of scattering and absorption. IFS scans were performed with excitation power of 1.5 mW and integrating time of 10 milliseconds. Hb concentrations in the two sets of replicate phantoms were determined from the DRS data and found to be 3.60±0.20 and 0.33±0.03 mg/mL. Figure 5A shows that the DRS spectra of the 4 phantoms vary primarily with the Hb concentration, whereas the IFS spectra shown in Figure 5B are largely independent of Hb concentration and depend on the concentration of furan. The results of these liquid phantom experiments confirm that the tissue scanner can accurately measure fluorophores in a variable background of absorption and scattering across a large scanning field of view.

**Table 1:** IFS tissue simulating phantom mixtures of furan, intralipid and blood.

	Furan (µg/mL)	Intralipid	Hb (mg/mL)
**Phantom 1**	0.8	1%	3.80
**Phantom 2**	0.8	1%	3.40
**Phantom 3**	0.3	1%	0.35
**Phantom 4**	0.3	1%	0.30

### Normal and cancer breast tissue imaging

**Figure 6:**
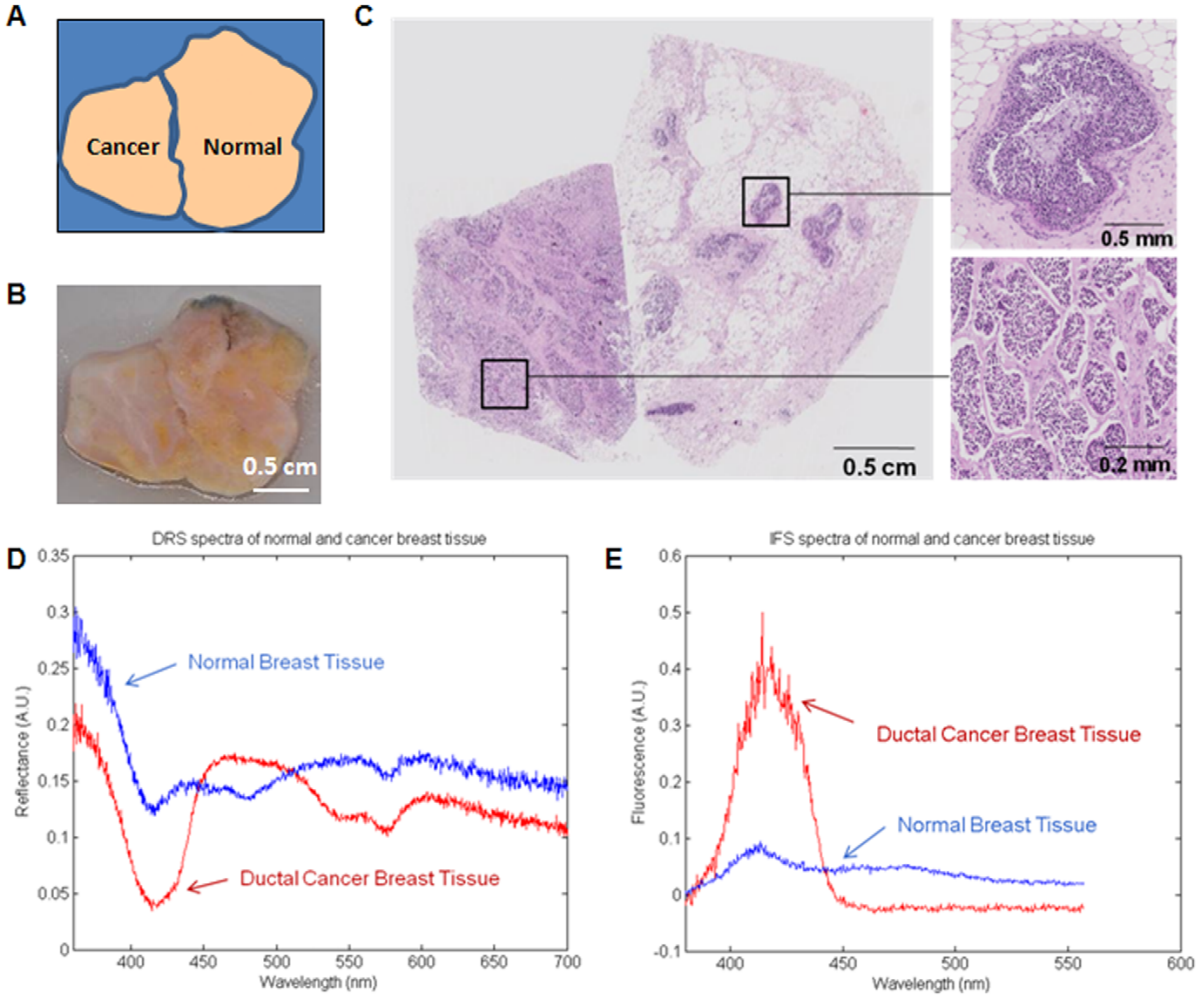
DRS and IFS of breast cancer and normal breast tissue. Diagram of normal and breast cancer tissues placed on glass plate during scanning (A); Gross photograph of breast tissues (B); Composite photomicrograph of histopathology of breast tissues (C)(top insert: ductal carcinoma *in situ*; bottom insert: invasive ductal carcinoma); DRS (D) and IFS (E) spectra of breast tissues.

To demonstrate the capability of the scanner to identify human breast cancer tissue, a paired set of frozen-thawed breast tissues, one grossly cancerous and another matched grossly normal breast tissue from the same patient, were placed side-by-side on the scanner glass plate as close together as possible to minimize the gap between the tissues (Figure 6A–B). DRS and IFS scans were performed by simultaneously scanning the 3 cm×3 cm region of interest, using separate DRS and IFS probes with a probe separation of 0.75 cm and spatial resolution of 0.25 mm per pixel. Excitation power and integrating time were 400 mW and 10 milliseconds for DRS, and 1.5 mW and 10 milliseconds for IFS scanning. Total image acquisition time for 150×150 pixels was 18 minutes. 2D quantitative DRS and IFS spectral intensity maps were created. The DRS and IFS spectra were then modeled and fitting parameters extracted to form 2D quantitative parameter maps for comparison with pathology.

**Figure 7:**
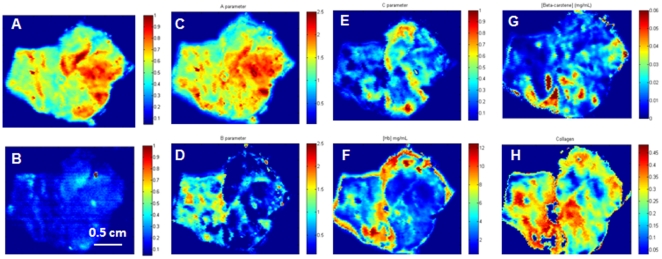
Spectral Intensity and Parameter Maps of Breast Tissue. DRS spectral intensity map of normal and cancer tissues at 545 nm (A); IFS spectral intensity map at 425 nm (B); DRS parameter maps for scattering parameters A, B and C, Hb and β-carotene, resp. (C–G); IFS parameter map for collagen (H).

Results of pathology examination (Figure 6C) confirmed that the grossly cancerous tissue consists largely of breast cancer (invasive ductal carcinoma). Interestingly, the grossly normal breast tissue, although seen microscopically to consist largely of normal breast tissue, contained multiple foci of breast cancer (ductal carcinoma *in situ*) <1 to 3 mm in diameter. DRS and IFS spectra obtained from scanner image pixels occupied by breast cancer and normal breast tissue (Figure 6D–E) are similar to those we obtained using similar optical fiber probes in a non-imaging multimodal spectroscopy system [15]. However, although individual 2D DRS and IFS spectral intensity maps demonstrate spectral contrast and have the requisite chemical information embedded in them, they do not by themselves reliably distinguish normal breast tissue from breast cancer (Figure 7A–B).

**Figure 8:**
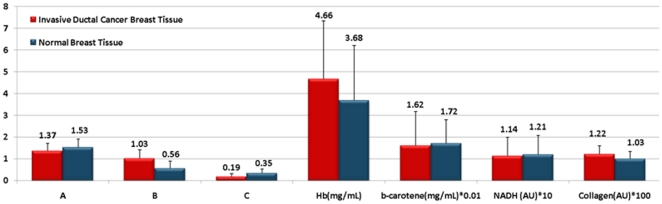
Fitting Parameters in Normal and Breast Cancer Tissue. Bar chart of pixel-to-pixel means DRS and IFS parameters in normal and breast cancer tissues.

To explicitly distinguish between cancerous and normal breast tissue, we employed the physico-chemical fitting parameters extracted from the DRS and IFS spectra. A total of seven parameters were extracted from the spectra and used to characterize the tissue: five DRS parameters (A, B, C, Hb and β-carotene) and two IFS parameters (collagen and NADH). Figure 8 is a graphical representation of the mean and standard deviation of these spectral parameters for normal and cancer breast tissues. The results show that the normal tissue has clearly different mean values of scattering, absorption and fluorescence parameters than the cancer tissue, and statistical t-test analysis shows that these differences are significant for all parameters at the 99% confidential level (Table 2). Specifically, higher values of A, C, β-carotene and NADH parameters are found in the normal breast tissue, whereas higher values of B, Hb and collagen parameters are found in the breast cancer tissue. The higher values of the C parameter are consistent with the increased size of tumor cell nuclei, and as a result nucleus-to-cytoplasmic ratio, characteristic of breast cancer (both invasive ductal carcinoma and ductal carcinoma *in situ*) [24]. The higher values of the Hb and collagen parameters are also consistent with the presence of angiogenesis and stromal fibrosis, resp., typically seen in breast cancer.

**Table 2:** T-test of the null hypothesis that means of normal and cancer DRS/IFS parameters are not equal.

Fitting Parameter	h (99%)	p Value	Confidence Interval	tstat Value	Degree of Freedom	Pooled Sample SD
**A**	1	0	[ 0.1872–0.2250 ]	28.1151	11496	0.3882
**B**	1	0	[−0.5644–(−0.5201) ]	−63.0668	8933	0.4062
**C**	1	0	[ 0.1453–0.1686 ]	44.3469	9623	0.1662
**Hb**	1	0	[−1.1695–(−0.7850) ]	−19.7833	11368	2.597
**β-carotene**	1	0	[ 0.0004–0.0017 ]	3.9941	10795	0.0129
**Collagen**	1	0	[−23.4554–(−19.79) ]	−30.3962	9845	34.4264
**NADH**	1	0.0079	[ 0.0281–1.8752 ]	2.6558	3462	8.695

**Figure 9:**
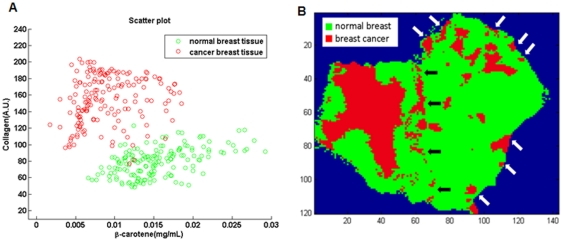
DRS-IFS Diagnostic Algorithm. Scatter plot of decision algorithm using DRS β-carotene and IFS collagen parameters for selective regions in normal and cancer breast tissues (A); Diagnostic map of normal and breast cancer tissues using the decision algorithm (B). Please note that: 1) regions denoted as cancer at the border of the grossly cancerous and normal tissues (white arrows) are most likely an artifact due to inadvertent gaps between the two tissues; and 2) regions denoted as cancer at the outer edges of the tissues (black arrows) are most likely due to the presence of black colloidal ink applied to the margin of the surgical specimen prior to harvesting for research use.

As with the DRS and IFS spectral intensity maps, the individual DRS and IFS parameter maps do not by themselves reliably distinguish normal breast tissue from breast cancer (Fig. 7 C–H). However, normal breast tissue could be reliably distinguished from breast cancer in the tissue scanner images by employing a decision algorithm based on a combination of DRS and IFS parameters, developed in our previous point probe study [15]. In this algorithm, the IFS collagen and DRS β-carotene parameters are used to distinguish normal breast tissue from all breast lesions including fibrocystic change, fibroadenoma and cancer. Since this experiment includes only normal and cancerous breast tissue, this same algorithm should provide adequate diagnostic discrimination, if it is transferable (i.e. is robust). The parameter scatter plot for the DRS β-carotene and IFS collagen parameters and diagnostic map based on these 2 parameters in Figure 9 show that the DRS-IFS algorithm is indeed transferable and is sufficient to distinguish the tissue types in this case. Representative data points were randomly selected from the two tissue regions for the parameter scatter plot shown in Figure 9A. The diagnostic map in Figure 9B identified not only the large focus of invasive ductal carcinoma in the grossly cancerous breast tissue (on the left), but also small (<1 to 3 mm) foci of ductal carcinoma *in situ* in the grossly normal breast tissue, which cannot be identified in the individual DRS β-carotene (Figure 7G) and IFS collagen (Figure 7H) parameter maps. This suggests that the tissue scanner has sufficient spatial resolution and spectral contrast to detect small foci of cancer in surgical margins. This proof-of-concept experiment lays the foundation for further work in more extensive clinical characterization of the instrument and its application to intraoperative assessment of surgical margins for cancer in the breast and other organ systems.

## Discussion

Here we describe a portable, quantitative, multimodal, optical fiber probe-based spectroscopic tissue scanner. The scanner's wide field of view, high resolution and quantitative hyperspectral imaging capability is demonstrated via a series of systematic experiments, namely in a resolution target, tissue-simulating phantoms, animal and human breast cancer tissue. Overall, theses studies show that the tissue scanner has sufficient spatial resolution and spectral contrast to detect small foci of cancer in surgical margins. Thus, this tissue scanner has the potential to provide timely, comprehensive, intraoperative assessment of surgical margins during cancer surgery. This can be accomplished by obtaining a diagnostic spectroscopic image of the surgical margin of the excised tissue specimen, identifying areas of the margin that are positive for cancer, and then relaying this information to the surgeon while he/she is still in the operating room and can remove additional marginal tissue if needed. This will allow more complete resection of diseased tissue and better conservation of normal tissue, and eliminate the need for a second surgery to achieve negative margins.

For solid tumors in organs such as the lung, liver and kidney, the surgical margin is only a small portion of one surface of the tissue specimen, and can easily be imaged in one pass in much less time than the 20 minutes required to scan an 8 cm×8 cm area using the current scanner configuration. In the special case of breast cancer, which we used as a test case here, the entire surface of the specimen must be imaged. As breast surgical specimens are generally relatively thin, roughly elliptical, soft and easily deformable, we should be able to image the entire margin by imaging two opposing surfaces of the ellipse. If necessary gentle pressure can be uniformly applied to the breast specimen, as is done during mammography, to insure full tissue surface contact.

One potential advantage of our tissue scanner is that it uses reflectance and fluorescence spectroscopy, which have tissue penetrations on the order of tens to hundreds of microns, and will thus give us the closest possible assessment of the true tissue surface margin. For most solid tumors, it is the status of this true margin that guides intraoperative management of tumor excision. In the special case of breast cancer, which we used as a test case here, intraoperative management of tumor excision is currently guided by near margins, that is, margins with tumor 1–5 mm below the tissue surface, which are used clinically as a surrogate for true positive margins. This is due to the sampling error inherent in histopathology margin assessment which, in the case of breast specimens, has an inherent bias towards false negative margins. In contrast, the tissue scanner is a high resolution imaging device that will scan the entire margin, is not subject to the sampling error inherent in histopathology margin assessment, and therefore has the potential to detect even the smallest focus of tumor at the true margin. Ultimately, the final judgment as to the clinical utility of true margin assessment using this, and any other imaging system will be based on clinical outcome, that is, whether spectroscopic margin assessment better predicts local recurrence than current histopathology techniques.

The design of our tissue scanner enables quantitative spectroscopic measurements without direct contact with the tissue (thereby enhancing its reproducibility), while maintaining a fixed tissue-probe geometry (which reduces extraneous variance from being incorporated in the measurement). It is a simpler design than those reflectance and fluorescence imaging systems designed for intraoperative margin assessment that utilize a complex array of multi-channel optical fiber probes [8] or an operating microscope for signal collection [9] or utilize a high resolution confocal microscope [7] or micro-endoscope [4] to achieve high spatial resolution. Finally, our tissue scanner combines DRS and IFS with spectral modeling to provide a more complete, quantitative assessment of the metabolic, biochemical and morphological state of tissue and, as a result, a more robust tissue diagnosis than can be achieved with either modality alone or by using the purely statistical algorithms for disease diagnosis currently used in some reflectance and fluorescence imaging systems.

Clinical translation will require more exhaustive evaluation of our current tissue scanner via larger scale intraoperative clinical studies of tissue specimens from patients with breast cancer and solid tumors in other organ systems. For the broadest possible clinical application, including intraoperative assessment of breast margins, it is desirable to improve imaging speed. Nevertheless, we believe that there is a significant need for a wide field, high resolution tissue scanner for intra-operative margin assessment that operates within the current temporal regime. In other words, while we plan to incorporate minor hardware and software changes to our instrument in the near future, the current system can be used for a wide variety of clinical investigations, particularly in solid tumors in organs other than the breast. Indeed, breast cancer margin assessment may represent the worst-case scenario with respect to time required for image acquisition, as the margins of breast surgical specimens are significantly larger than the margins of surgical specimens from other organs. These surgical margins are often less than 7.5 cm×5 cm in area and can be imaged in less than 5 minutes with the improvements. Clearly, smaller surgical margins in organs other than the breast considerably reduce the time required for spectral acquisition and image analysis.

Never-the-less, in order to further reduce image acquisition time, we plan to incorporate both hardware and software changes. Currently, only one of the collection fibers in the optical fiber probe is coupled to the spectrograph. Prior to embarking on large scale clinical studies, we plan to couple all 6 collection fibers available in the existing optical fiber probe to the spectrograph. The circular array of collection fibers surrounding the excitation fiber at the proximal end of the probe will be converted into a linear array at the distal end of the probe, much as in our other clinical spectroscopy systems [10]–[11], which will align with the fiber array on a simple 2-D spectrograph with built-in CCD. With this configuration, we can image up to 6 separate tissue sites simultaneously. This change will not significantly increase instrument complexity or cost, and will provide up to a 6-fold reduction in imaging time. Further, we will incorporate state-of-the-art chemometrics and machine learning algorithms to allow real time processing of the spectral data. These hardware and software strategies combined should significantly reduce the time required to scan the margins of surgical specimens, render a diagnosis of margin status (positive or negative) and provide feedback to the surgeon. Finally, while our current tissue scanner provides powerful hyperspectral capabilities with spectral resolution of ∼2 nm, such resolution may not be necessary for all cancer diagnostics applications. Relaxation of the spectral resolution constraint can give rise to interesting design possibilities focusing on particular spectral features of interest. Such an approach is likely to lead to a substantial reduction in imaging time, instrument size and the overall cost of future tissue scanner systems.

We anticipate that the current tissue scanner system can be readily extended to provide real-time comprehensive assessment of surgical margins in excised tissue specimens, overcoming the sampling limitation in current histopathology margin assessment. This device is an important step in the development of a platform technology for intraoperative management of cancer, a clinical problem that has been inadequately addressed to date.
